# Application of a Large Visual Language Model on Tongue Image Description Generation and Physical Constitution Reasoning in Traditional Chinese Medicine (TongueVLM): Model Development and Validation Study

**DOI:** 10.2196/87237

**Published:** 2026-03-12

**Authors:** Chengdong Peng, Jun Gao, Nuo Yang, Yong Wang, Renming Chen, Changwu Dong

**Affiliations:** 1 School of Computer Science and Information Engineering Hefei University of Technology Hefei, Anhui China; 2 Artificial Intelligence Laboratory Hefei Yunzhen Information Technology Co, Ltd Hefei, Anhui China; 3 School of Information and Artificial Intelligence Anhui Agricultural University Hefei, Anhui China; 4 The Second Clinical Medical School Anhui University of Traditional Chinese Medicine Hefei, Anhui China

**Keywords:** vision-language model, tongue image description generation, visual constitution reasoning, visual dialogue, intelligent diagnosis of traditional Chinese medicine (TCM)

## Abstract

**Background:**

In the field of traditional Chinese medicine (TCM), diagnostic work based on tongue images to recognize the physical constitution is a process of collecting clinical information, reasoning, and combining the patient’s tongue image features with questioning. It is necessary to simulate the recognition of pathological information of tongue images by TCM practitioners and professional dialogue based on tongue image features, which helps to develop an intelligent interactive system for TCM diagnosis.

**Objective:**

This study aimed to develop and validate a vertical model of the TCM domain with TCM’s understanding and reasoning capability for tongue images.

**Methods:**

A TongueVLM multimodal large model is designed, which includes a visual encoder module, a modal fusion module, and a language decoder module. First, the visual encoder based on the CLIP-ViT (Contrastive Language-Image Pre-Training With Vision Transformer) pretrained model is used for image patch, dimensionality reduction, and migration learning, which maps the high-dimensional tongue features into low-dimensional language encoding vectors. Further, a modal fusion module with a residual architecture is applied to map visual features to a natural language word embedding space, realizing the conceptual alignment between visual encoding and TCM terminology. Finally, fine-tuning of visual instructions is performed based on the LLaMA (large language model meta artificial intelligence), and a TCM-domain large language model with 7B parameters is trained.

**Results:**

The constructed multimodal dataset has 3 test datasets, and experiments are conducted using 3000 samples from each test dataset, respectively. Experimental results indicate that the TongueVLM model outperforms general-purpose large models on all 3 tasks. On the multimodal test dataset, the TongueVLM model achieved accuracy rates of 79.8%, 78.6%, and 60.7% in evaluation tasks respectively, it achieves 9.1%, 8.4%, and 1.1% in greater accuracy than LLaVA-OneVision, and is 7.5%, 7%, and 5.9% more accurate than Qwen2.5-VL-7B, with the text generation time being around 24 tokens per second.

**Conclusions:**

The TongueVLM model, which achieves tongue image description generation and physical constitution reasoning in TCM, is suitable for the application of a Chinese medicine intelligent diagnosis system.

## Introduction

In recent years, the research focus on computer vision has gradually shifted toward innovative transformers and their variant architectures. These studies have made significant progress in the 3 basic tasks of image classification, object detection, and image segmentation, along with in-depth experiments on visual-textual multimodal data validity. The visual transformer model, represented by the vision transformer, approaches or even surpasses the convolutional neural network approach in several benchmark tests [1-14]. The key technique of the vision transformer is to construct image-to-vector transformations and effectively maintain the features of the image, bridging the gap between language and vision. Lava family [15-17], GPT-4v [18], and Gemini 1.5 [19] visual-language multimodal modeling have even ushered artificial intelligence-generated content‌ into a new era of visual applications, and the field of medicine has received increasing attention from researchers [20-26].

In the field of traditional Chinese medicine (TCM), diagnostic work based on tongue images to recognize the physical constitution is a process of collecting clinical information, reasoning, and combining the patient’s tongue image features with questioning. It is necessary to simulate the recognition of pathological information of tongue images by TCM practitioners and professional dialogue based on tongue image features, which helps to develop an intelligent interactive system for TCM diagnosis. However, the following limitations still exist in the current investigation: (1) high cost of acquisition equipment and collection of clinical data, mostly relying on publicly available datasets, and prominent data sample noise problems; (2) lack of qualitative or quantitative specialized labeling data; (3) lack of validation and analysis of the visual encoding results at the modal fusion stage; and (4) the evaluation metrics of the trained models focus on technical performance and ignore professional human judgments.

Previous studies have shown that, based on the image recognition model in the field of TCM applications, they fused the traditional image features of the tongue with deep tongue features and combined them with a machine learning model to construct a physical constitution identification, which compensated for the lack of information in a single modality and improved the accuracy of constitution classification [27-29]. In terms of training a vertical model of TCM based on a large language model (LLM), we constructed a pretraining dataset and an instruction fine-tuning dataset oriented to TCM and adopted a 2-phase training method of continuous pretraining and supervised fine-tuning to develop an LLM of TCM [30-35]. This model outperforms the existing models in several evaluation metrics and can play a role in TCM consultation.

Medical applications based on multimodal large models are also favored by researchers. A dataset containing 5.3 million+ image-text pairs was used to train the UniMed-CLIP model, which outperforms the existing health care visual language models (VLM) with 0-shot performance [36]. Multimodal health care models, which introduce evaluation metrics and frameworks and perform well in multimodal Q&A (question and answer) and text-based Q&A tasks [33,37]. BiomedCLIP with LLaMA-3 (large language model meta artificial intelligence) to realize a lightweight and efficient medical visual Q&A model [38]. Med-VQA interpretability by localizing image preference regions [39] enhanced the performance of biomedical VLMs by using instruction fine-tuning and multi-image training, respectively [40,41]. Aligned visual features with medical concepts and Med-VQA, enhanced the generation of large models, and achieved excellent performance on public datasets [42-44].

The primary aim of this study is to develop and validate TongueVLM, a specialized VLM for TCM tongue diagnosis. Specifically, our objectives were to (1) design a novel multimodal architecture that effectively aligns visual features of tongue images with domain-specific TCM textual knowledge; (2) construct a high-quality, multimodal dataset for supervised study, and end-to-end training on the TCM dataset; and (3) evaluate the performance of TongueVLM against established baseline models, and validation multimodal capabilities across 3 tasks of tongue feature description generation, physical constitution reasoning, and multiround dialogue.

## Methods

### Overall Architecture of TongueVLM

**Figure 1:**
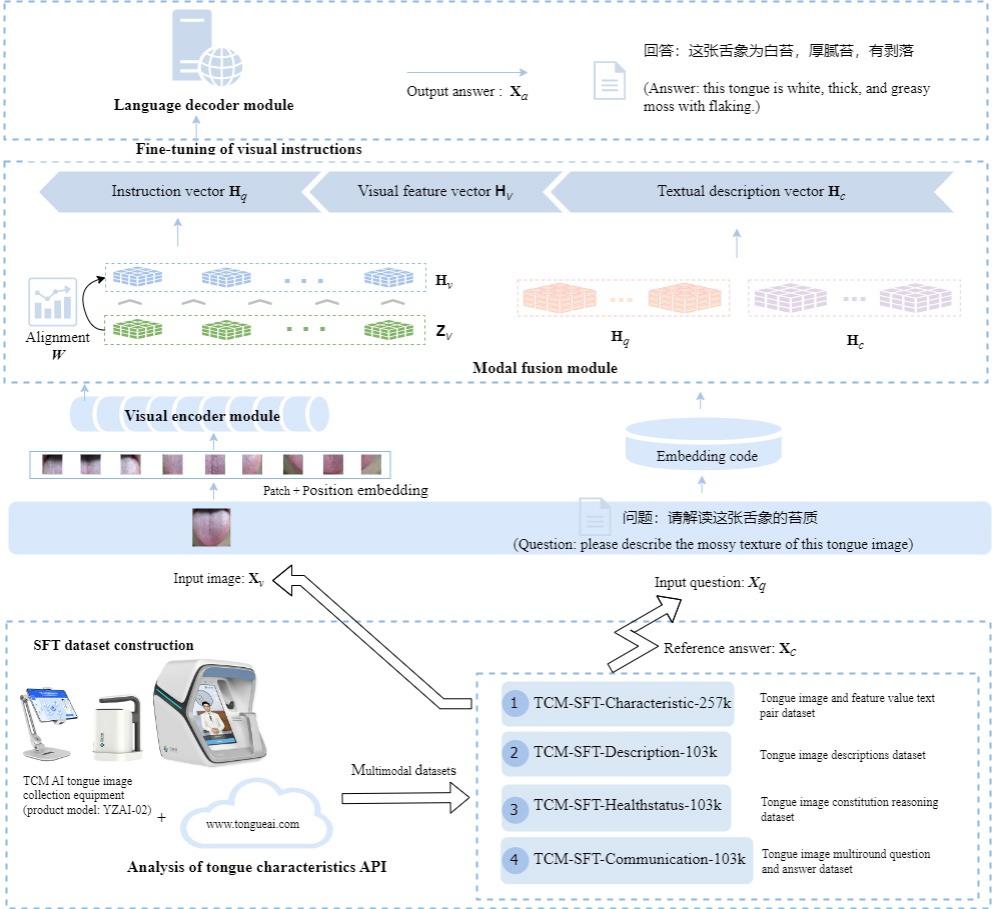
Network structure and computation of a large visual-language model. Includes a visual encoder module, a modal fusion module, and a language decoder module. First, the visual encoder based on the CLIP-ViT pretrained model is used for image patch, dimensionality reduction, and migration learning. Further, a modal fusion module with a residual architecture is applied to map visual features to a natural language word embedding space. Finally, fine-tuning of visual instructions is performed based on the LLaMA. AI: artificial intelligence; API: application programming interface; CLIP ViT: Contrastive Language Image Pre Training With Vision Transformer; LLaMa: large language model meta artificial intelligence; SFT: supervised fine-tuning; TCM: traditional Chinese medicine.

Our TongueVLM model uses a hybrid architecture combining the visual encoder module, modal fusion module, and language decoder module. First, the visual encoder based on the CLIP-ViT (Contrastive Language‑Image Pre‑Training With Vision Transformer) pretrained model is used for image patch, dimensionality reduction, and migration learning. Further, a multimodal fusion module with a residual architecture is applied to map visual features to the natural language word embedding space, realizing the conceptual alignment between visual encoding and TCM terminology. Finally, a visual-textual instruction dataset is constructed to perform multimodal instruction fine-tuning based on the LLaMA general-purpose LLM. As a result, a vertical model of the TCM domain with TCM understanding and reasoning capability for tongue images is obtained. The realization procedure is shown in Figure 1.

### Model Design

#### Phase 1: Visual Encoder Module

In the first phase, the visual encoder module of the network was designed. Applying tongue images with TCM feature description text pair data in CLIP-ViT pretrained model migration learning. Semantically rich text is used as training labels to project the image feature representations into a kind of encoding that is like the text encoding space, as shown in Figure S1 in Multimedia Appendix 1.

First, we speak of image patch dimensionality reduction preprocessing. Scale the input image **x***_v_*∈(*H*,*W*,*C*) to **x**∈(*h*,*w*,*C*) (h is the height, w is the width, and C is the number of channels). Sequence of blocks that transform x into **x***_p_*∈(*N*^2^,*p*^2^ *C*) for image patch operations. The number of blocks is *N*^2^=(h×*w*)/*p*^2^, and the block dimension is (*p*^2^ *C*); where *p* is the image patch size. A sequence of blocks is converted into a patch embedding vector **E***_patc_*_h_^'^∈(*N*^2^,*dim*) by a linear transformation, where *dim*=1024, *p*=14, and *N*= 24. ***x***_0_ is the zeroth encoding vector taken at the output layer of the transformer encoder, which serves as the visual feature encoding. By adding a learnable embedding vector before the image patch embedding vector E_patch_, the image block embedding vector becomes **E***_patc_*_h_∈(*N*^2^+1,*dim*). The absolute position encoding algorithm is used to create a learnable position embedding vector **E***_pos_*∈(*N*^2^+1,*dim*) for the block position. The elementwise summation operation on **E***_patc_*_h_ and **E***_pos_* yields an embedding vector **z**∈(*N*^2^+1,dim) that incorporates spatial positional encoding and visual representations.

Second, transformer encoder processing. The encoder consists of a stack of **L**=24 encoding layers, and the encoder inputs are image feature embedding vectors *z*∈(*N*^2^+1,*dim*). Each encoding layer consists of 2 unitary structures connected: the first unitary structure consists of a multihead self-attention layer and a layer normalization, as well as a residual connectivity module, and the second unitary structure consists of a feedforward fully connected layer multilayer perceptron and a layer normalization, as well as a residual connectivity module.

#### Phase 2: Modal Fusion Module

The modal fusion module is designed with a multilayer linearly transformed residual architecture, which is a bridging module between the visual model and the language generation model. The tongue visual encoding features are projected into the natural language word embedding vector feature space to solve the conceptual alignment problem of visual encoding with TCM terminology.

The modal fusion module design uses a combination of multiple linear layers and GELU (Gaussian Error Linear Unit) activation functions, as shown in the middle region of Figure S2 in Multimedia Appendix 1. This design allows the modal fusion layers to transform visual features while maintaining essential nonlinear characteristics. In addition, the introduction of the residual structure enables the model to realize efficient feature transformation and projection while fully preserving the integrity of the original visual features.

Splice visual encoding with textual word-embedded encoding to form a sequence of visual-textual instructions. First, the visual encoding feature **Z***_v_* is converted into a visual feature vector **H***_v_*, and the textual instruction and textual description are converted into an instruction vector **H***_q_* and a textual description vector **H***_c_* by word embedding encoding, respectively. Thereafter, the vectors are spliced in the order of instruction, visual, and textual description to form the visual-textual fusion instruction sequence data.

#### Phase 3: Language Decoder Module

The modal fusion module connects the visual encoder and language decoder for end-to-end fine-tuned training on the constructed visual-textual data for a variety of downstream generative output tasks, such as visual quizzing, image description generation, and visual dialogue. The language decoder module chooses LLaMA, a pretrained model with good support for Chinese, which consists of a 32-layer transformer decoder layer, and each decoder layer contains an RMSNorm normalization layer, a grouping attention mechanism, and a multilayer perceptron projection layer. The network architecture is shown on the right side of Figure S2 in Multimedia Appendix 1.

During training, each input image **X***_v_* is associated with a few rounds of dialogue data, where each round of question description 
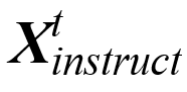
 can be defined as follows.



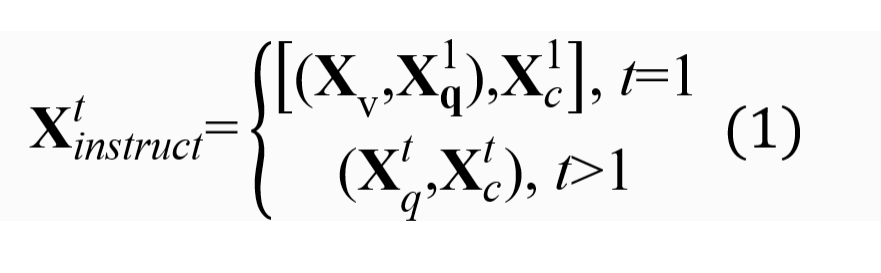



The visual encoder module extracts the visual feature V_x_ (the feature representation obtained by encoding the input image using a visual encoder) and caches it into the context, and the subsequent t-th round (t>1) of training data only needs to input the new instruction 
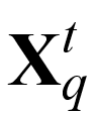
 and answer 
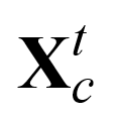
. The model automatically fuses the historical dialogue information with the initial visual feature **V***_x_* and builds a spliced sequence of instructions 
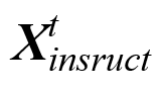
 to realize multiple rounds of dialogue training through cross-round dependency.

The instruction sequence 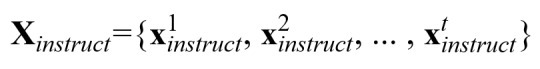 of multiple rounds and the corresponding output reply text 
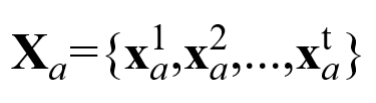
 are temporally spliced into a uniform sequence 
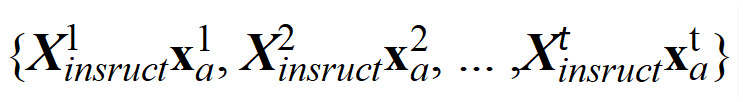
, the autoregressive training objective is used for instruction tuning, and the maximum likelihood function optimization model is formulated as follows:



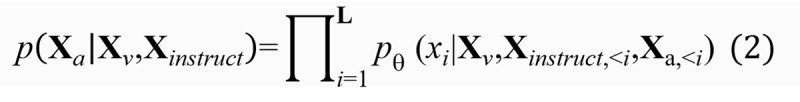



where θ is a trainable parameter (the training process is to adjust to maximize the likelihood function) and **X***_instruct,<i_*,**X***_a,<i_* is the instruction sequence and answer sequence of all rounds before the current prediction *x_i_*, respectively. *p*(**X***_a_*|**X***_v_*,**X***_instruct_*) is the probability of predicting **X***_a_* under the current **X***_v_* and **X***_instruct_* is based on the premise of guaranteeing the accuracy of the result of **X***_a_*under the premise that the prediction results of all rounds before the current round takes the maximum probability value.

In other words, the model uses the information accumulated in the history of the previous *t*-1 rounds of the conversation (especially the context of the textual instructions) to accurately estimate the probability distribution of the generated results in the t-th round. In this way, the model can maintain its understanding of the context over consecutive rounds of interactions and accordingly generate accurate responses associated with the content of the previous rounds.

#### Datasets and Preprocessing

The 90,000 tongue images collected by the TCM artificial intelligence tongue image collection equipment (product model: YZAI-02) jointly developed by Hefei Cloud Diagnostics Information Technology Co and Anhui University of TCM were used, which consists of a standard LED ring light source, a portable collection device, an ultrahigh-definition camera, and a host computer, as well as supporting software. The tongue feature recognition API (application programming interface) was applied to preprocess the collected tongue images with color correction, tongue region segmentation, and tongue moss and tongue texture separation, after which tongue feature analysis was performed on the tongue images to obtain more than 40 types of qualitative or quantitative text containing tongue color, tongue texture, moss color, moss texture, and sublingual collateral data and 10 types of TCM constitution [45].

The TongueVLM training datasets are constructed, including TCM feature description data (traditional Chinese medicine–supervised fine-tuning, TCM-SFT (TCM–supervised fine-tuning)-Characteristic-257k, and TCM-SFT-Description-103k), multiround Q&A data (TCM-SFT-Healthstatus-103k), and physical constitution reasoning data (TCM-SFT-Communication-103k). The methodology for constructing the dataset is described in Multimedia Appendix 1. The distribution of the tongue image features in the dataset is shown in Figures S3 and S4 in Multimedia Appendix 1.

### Model Training Strategy

#### Stage 1: Pretraining of the Visual Encoder

TCM tongue characterization datasets (TCM-SFT-Characteristic-257k) of 257,000 tongue images and characterization texts are constructed, and the zeroth encoding vector used for learning by comparing the similarity between the tongue images and their corresponding tongue manifestation characterization is used for training the fine-tuning of the tongue visual encoder.

**Figure 2:**
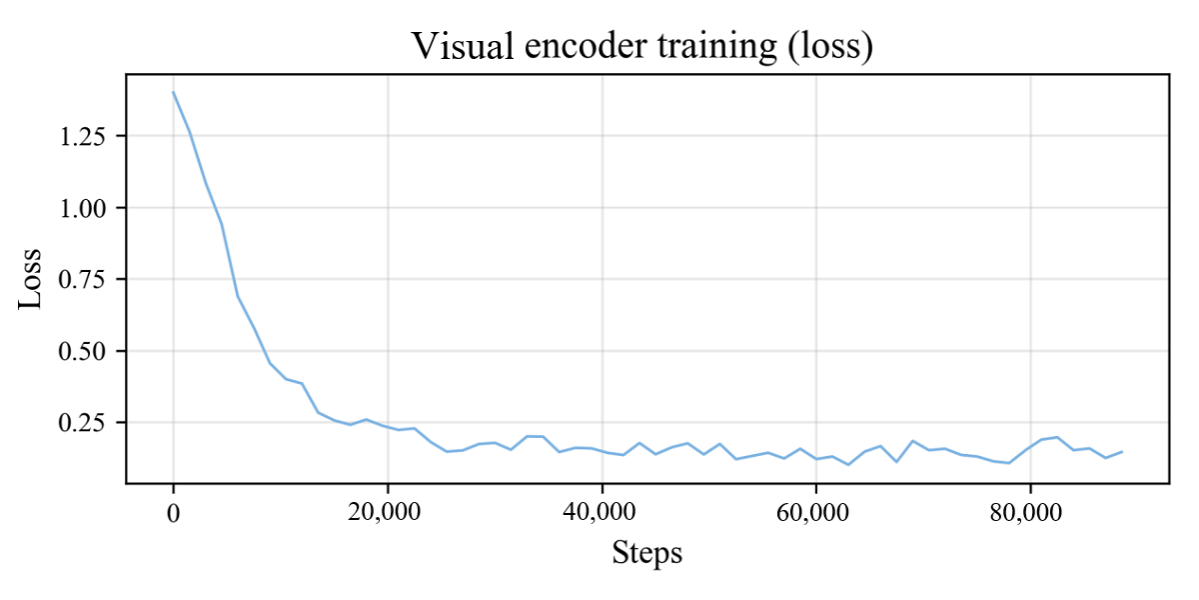
Visual encoder training loss and dynamic learning rate curves. The loss curve shows that after 3 rounds of training, the training loss of modal fusion has gradually stabilized, indicating that the model is beginning to converge.

The process to obtain the visual encoder model is as follows: first, the training parameters openai/clip-vit-large-patch14-336 (OpenAI Inc, pretrained weights for the clip model on 400 million text-image pairs) were used as the initial weights for the visual encoder and the text transformer encoder. The Adam optimizer was used in the model training, with the smoothing constant betas set to (0.9, 0.999) and the weight decay parameter weight_decay set to 1e-3, the training batch size was set to 8, the learning rate was 5e-5, the number of training epochs was 3, and the maximum sequence length was 77. Second, the loss function uses the cosine similarity Cos(I_m_,T_n_),m∈[1…N^2^],n∈[1…N^2^], where I_m_ is the embedding vector encoded by visual features and T_n_ is the embedding vector encoded by textual semantics. The optimization objective is to maximize the cosine similarity Cos(I_j_,T_j_) between correct image-text pair embeddings while minimizing the cosine similarity Cos(I^j^,T^k^),j≠k of incorrect image-text pair embeddings. The results show that the loss value stabilizes after 3 epochs of training, and the loss curves are shown in Figure 2.

#### Stage 2: Training of the Fusion Modal

TCM tongue description datasets (TCM-SFT-Description-103k) of 103,000 tongue images and description text are constructed, the visual encoder of TongueVLM and the network weights of the LLM are frozen, the visual-textual multimodal instruction data are fed into the LLM, and the modal fusion module is fine-tuned for training.

**Figure 3:**
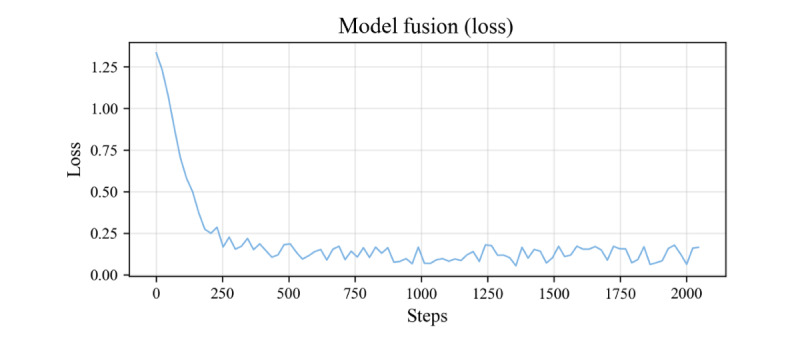
Loss curve of the modal fusion model. It shows that after 3 training epochs, the model enters a stable convergence state, and the loss curve for its modal fusion training stabilizes.

The process to obtain the modal fusion model is, first, the training method and parameters are as follows: the pretrained visual encoder weight parameters are loaded, the modal fusion layer weight parameters are randomly initialized, and only the modal fusion layer weights are allowed to participate in the gradient update. The Adam optimizer was used in the model training, with the smoothing constant betas set to (0.9, 0.999) and the weight decay parameter weight_decay set to 1e-4, the training batch size on each GPU is 8, the gradient accumulation step is 8, the learning rate is 2e-5, and the total number of iteration rounds is 3. Spend 30 hours to complete all the training on the Ubuntu (version 20.04.6, Canonical Ltd) platform using 2×RTX4090. Second, the loss function adopts the cross-entropy loss 
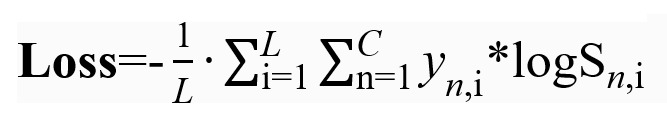
 , where *C* is the total number of word lists, *L* is the length of the prediction sequence, S*_n,i_* denotes the probability that the word in the ith position is the nth word in the lexicon based on the previously known sequence, and y_n,i_ denotes the ground-truth labels in the ith position in the prediction sequence (expressed as 1-hot encoding; ie, if the corresponding word in the position is the nth vocabulary word in the lexicon, only the first element of the 1-hot vector is 1 and the rest are 0), the goal is to minimize the loss; when the probability of each word in the prediction sequence is infinitesimally close to the actual vocabulary 1-hot encoding, that is, for any position *i* in the prediction sequence, S_n,i_≈y_n,i_, the loss will be minimized, and at this time, the model is considered to be in the optimal state. The results show that the loss value stabilizes after 3 epochs of training, and the loss curves are shown in Figure 3.

#### Stage 3: End-to-End Fine-Tuning of TongueVLM

**Figure 4:**
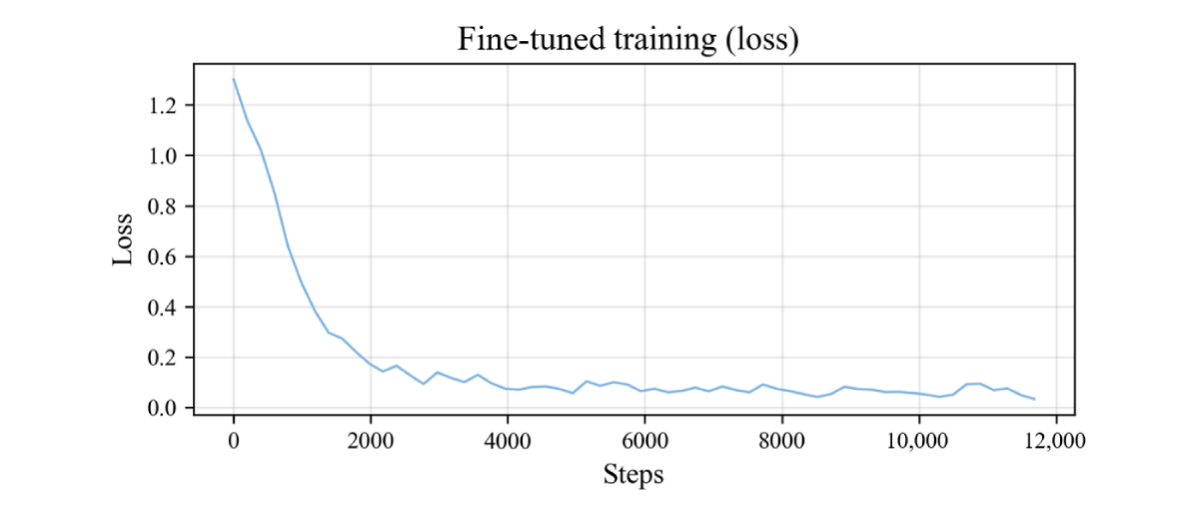
Fine-tuned training loss curve of TongueVLM. It shows that with the increase of training epochs, the fine-tuning training loss of TongueVLM gradually tends to stabilize, indicating the effectiveness of the optimization algorithm and the basic convergence of the model after about 8000 iterations.

The training data are the TCM multimodal dataset (training set 510,000; validation set 25,000). First, of the training method and parameters, load the pretrained visual encoder weight parameters and modal fusion layer weight parameters, freeze the weight parameters of the visual encoder, and allow only the modal fusion layer and the language decoder weights to participate in the gradient update. The Adam optimizer was used in the model training, with the smoothing constant betas set to (0.9, 0.999) and the weight decay parameter weight_decay set to 1e-4, the training batch size is set to 8 on each GPU, the number of gradient accumulation steps is 8, the learning rate is 2e-5, and the total number of iteration rounds is 3. It takes 135 hours to complete all the training on the Ubuntu (version 20.04.6, Canonical Ltd.) platform using 2×RTX4090. Second, of loss function, the cross-entropy loss function is adopted, and the loss curve is shown in Figure 4. After 4000 iterations, the loss tends to stabilize, and the loss fluctuation is very small when the number of iterations is 8000; currently, the model has converged.

### Ethical Considerations

This study was conducted in accordance with the ethical standards of the Declaration of Helsinki and was approved by the ethics committee of the Second Clinical Medical School of Anhui University of Chinese Medicine (Anhui Acupuncture and Hospital). The data used in this research were sourced from 2 distinct origins. First, for data from the Yunzhen 365 app, the data consisted of deidentified user‑uploaded tongue images and associated text. All users of the app provided informed consent upon registration, agreeing to the use of their anonymized data for research purposes in accordance with the app’s terms of service and privacy policy. Second, for data from hospital records, the clinical data were obtained with approval from the relevant hospital’s institutional review board (2022-zjks-25). Patient confidentiality was strictly maintained, and all personal identifiers were removed before analysis.

All procedures involving human data were reviewed and approved by the appropriate ethics committees. No additional identifiable personal information was accessed or used in this study. Participants received no compensation for their involvement in this study.

## Results

### Overall Performance of TongueVLM on Benchmark Tasks

The performance evaluation of the TongueVLM on the TCM multimodal test dataset is as follows.

To determine whether the answer texts generated by professional TCM practitioners are close in meaning to the standard answer texts in the corresponding test set, the evaluation is divided into 3 results; according to the degree of similarity in meaning, we define a score value Score for it, in descending order: consistent (Score=100), similar (Score=50), and irrelevant (Score=0); the accuracy of TongueVLM on the test set can be expressed as follows: 
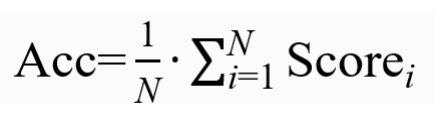
, where *N* is the number of test datasets and Score_i_ is the score of the *i-*th test dataset.

To directly verify the effectiveness of the improved methods proposed in this study for the field of TCM, we selected visual multimodal models with similar architectures for comparison: LLaVA-1.5 (released in October 2023; Meta Platforms, Inc), LLaVA-1.6 (released in January 2024; Meta Platforms, Inc), and LLaVA-OneVision (released in August 2024; Meta Platforms, Inc). To demonstrate that good results can be achieved in the field of TCM through targeted model design without relying on massive general data, we also compared it with the current open-source multimodal large model benchmark Qwen2.5-VL-7B (released in August 2024; Alibaba Cloud). The accuracy and performance are shown in Table 1. The experimental results show that the TongueVLM model outperforms the general-purpose large model on all 3 tasks; it is 9.1%, 8.4%, and 1.1% more accurate than LLaVA-OneVision and 7.5%, 7%, and 5.9% more accurate than Qwen2.5-VL-7B. The generation time efficiency of each large model is similar, with the generation time of text being 24 tokens per second (approximately 40 words per second, given the word-to-token ratio in our dataset). Due to the optimization of the lexicon in the field of TCM, TongueVLM inference speed is slightly faster than the baseline model.

**Table 1:** Evaluation indicators for the output of the model. Shows the evaluation metrics of the model output. Experiments are conducted using the test dataset of 3 multimodal datasets, with each test dataset containing 3000 samples with detailed data represented as: number of scores 0, number of scores 50, number of scores 100/total number of test datasets.

Model	TCM-SFT^a^-Description-103k			TCM-SFT-Healthstatus-103k				TCM-SFT-Communication-103k		
	Test result details	Accuracy (%)	Mean time (SD, s)	Test result details	Accuracy (%)	Mean time (SD,s)	Test result details		Accuracy (%)	Mean time (SD,s)
CNN^b^ parallel computing	167,58,2775/3000	79	1.48 (>6)	—^c^	—	—	—		—	—
LLaVA-1.5	1730,973,297/3000	26.1	0.27 (1.25)	1828,920,252/3000	23.7	0.24 (1.5)	1174,1653,173/3000		33.3	0.64 (3)
LLaVA-1.6	568,1285,1147/3000	59.7	0.21 (1.55)	545,1241,1214/3000	61.2	0.21 (1.5)	690,1094,1216/3000		58.8	0.62 (3)
LLaVA-OneVision	393,974,1633/3000	70.7	0.17 (1.54)	423,941,1636/3000	70.2	0.25 (1.5)	635,1156,1209/3000		59.6	0.86 (3)
Qwen2.5-VL-7B	412,836,1752/3000	72.3	0.18 (1.48)	420,866,1714/3000	71.6	0.15 (1.4)	519,1672,809/3000		54.8	0.69 (3)
TongueVLM	305,599,2096/3000	79.8	0.11 (1.25)	353,577,2070/3000	78.6	0.20 (1.5)	428,1502,1070/3000		60.7	0.54 (3)

^a^TCM-SFT: traditional Chinese medicine–supervised fine-tuning.

^b^CNN: convolutional neural network.

^c^Not available.

### Ablation Studies on the 3-Stage Training Strategy

#### Visual Encoding Feature Layer Comparison Experiment

The visual encoder extracts image features and outputs a total of 24 hidden layers (disregarding the hidden layer feature values before being input into the visual encoder), and it is necessary to select the appropriate layer as the visual feature information input. Therefore, comparison experiments were conducted on 3 datasets, and the last 5 hidden layers (20th to 24th) features were selected as the visual feature information to train the TongueVLM model; the accuracies on the test datasets are shown in Table 2 below. The experimental results show that the model performs best in all tasks when the penultimate layer of features output from the visual encoder, that is, the 23rd layer of features, is selected as the final visual feature information. These findings reveal that the visual features extracted at that depth level have significant advantages for multimodal intelligent analysis related to TCM tongue diagnosis.

**Table 2:** Accuracy of different hidden layers as a visual encoder in the test dataset. A total of 500 samples were randomly selected from each of the 3 multimodal test datasets for experimentation. It shows the accuracy of different hidden layers as visual encoders in the test dataset, with detailed data represented as: number of scores 0, number of scores 50, number of scores 100/total number of test datasets).

Hidden layers	TCM-SFT^a^-Description-103k		TCM-SFT-Healthstatus-103k		TCM-SFT-Communication-103k	
	Test result details	Accuracy (%)	Test result details	Accuracy (%)	Test result details	Accuracy (%)
Layer-24	54,102,344/500	79	44,29,427/500	88.3	60,29,411/500	85.1
Layer-23	51,100,349/500	79.8	44,26,430/500	88.6	60,23,417/500	85.7
Layer-22	52,105,343/500	79.1	45,37,418/500	87.3	63,25,412/500	84.9
Layer-21	52,114,334/500	78.2	45,49,406/500	86.1	63,32,405/500	84.2
Layer-20	55,117,328/500	77.3	48,48,404/500	85.6	65,47,388/500	82.3

^a^TCM-SFT: traditional Chinese medicine–supervised fine-tuning.

#### Modal Fusion Module Structure Experiment

To verify the reasonableness of the structure of the modal fusion module, 3 different neural network structures, namely, numbers 1 (Linear + GELU), 2 (Linear + GELU + Linear + GELU + Linear), and 3 (Linear + GELU + Linear + GELU + Linear + Linear + Res), are designed, and initial weights are randomized with the same datasets; the accuracy on the test datasets is shown in Table 3. The results show that the difference in the effect of the 3 structures on the final performance is not significant. However, the use of the fusion structure of group number 3, containing multilayer linear transformation, together with a multilayer nonlinear activation function and the introduction of residual connections, slightly outperforms the performance of the other schemes. Therefore, group number 3 is chosen as the neural network structure for modal fusion learning.

**Table 3:** Comparative analysis of the modal fusion neural network structure in terms of accuracy on the test datasets. A total of 500 samples were randomly selected from each of the 3 multimodal test datasets for experimentation with detailed data represented as: number of scores 0, number of scores 50, number of scores 100/total number of test datasets.

Number	TCM-SFT^a^-Description-103k		TCM-SFT-Healthstatus-103k			TCM-SFT-Communication-103k	
	Test result details	Accuracy (%)	Test result details	Accuracy (%)	Test result details		Accuracy (%)
1	55,99,346/500	79.1	46,26,428/500	88.2	62,27,411/500		84.9
2	54,98,348/500	79.4	45,29,426/500	88.1	60,27,413/500		85.3
3	51,100,349/500	79.8	44,26,430/500	88.6	60,23,417/500		85.7

^a^TCM-SFT: traditional Chinese medicine–supervised fine-tuning.

#### Modal Fusion Module Pretraining Experiment

The weight parameters of the modal fusion are also fine-tuned for training during the overall training of the TongueVLM model; thus, the pretraining of the modal fusion is compared to that of number 1, the pretraining is fine-tuned individually, and number 2 is randomly initialized in TongueVLM without pretraining. The accuracy of the 3 test datasets is shown in Table 4 below. The results show that the accuracy improvement using the individually fine-tuned pretraining approach is significant, with 2.3%, 5%, and 3.5% improvement for the 3 datasets, respectively. Fine-tuning the modal fusion layer using the Chinese interpretation feature description data allows the model to focus more on constructing and optimizing higher-order correspondences between visual-language features.

**Table 4:** Evaluation of pretraining methods for modal fusion modules. A total of 500 samples were randomly selected from each of the 3 multimodal test datasets for experimentation with detailed data represented as: number of scores 0, number of scores 50, number of scores 100/total number of test datasets.

Numbers	TCM-SFT^a^-Description-103k		TCM-SFT-Healthstatus-103k		TCM-SFT-Communication-103k	
	Test result details	Accuracy (%)	Test result details	Accuracy (%)	Test result details	Accuracy (%)
1	51,100,349/500	79.8	44,26,430/500	88.6	60,23,417/500	85.7
2	53,119,328/500	77.5	46,73,381/500	83.5	63,52,385/500	82.2

^a^TCM-SFT: traditional Chinese medicine–supervised fine-tuning.

#### Analysis of Module Effectiveness and Interpretability

In this experiment, the visualization of the projection of the attention weight values of the multihead self-attention layer during the forward computation of the visual encoder to the image is investigated, and pseudo-color maps of the regional thermal response during the visual encoding process for typical texture feature locations on the surface of the tongue image are generated.

The multihead attention weights during the forward propagation of the cached visual encoder are Atten_weights∈R^(num_heads,dim,dim)^, where num_heads∈(1,2,…,16) is the number of heads of multihead attention, and *dim*=577 is the length of the encoding sequence. The 0th position of the encoding sequence is the classification head, and the E_i_∈R^(dim–1,dim–1)^ vector represents the correlation between the 24 × 24 patches of the image in the ith attention header.

**Figure 5:**
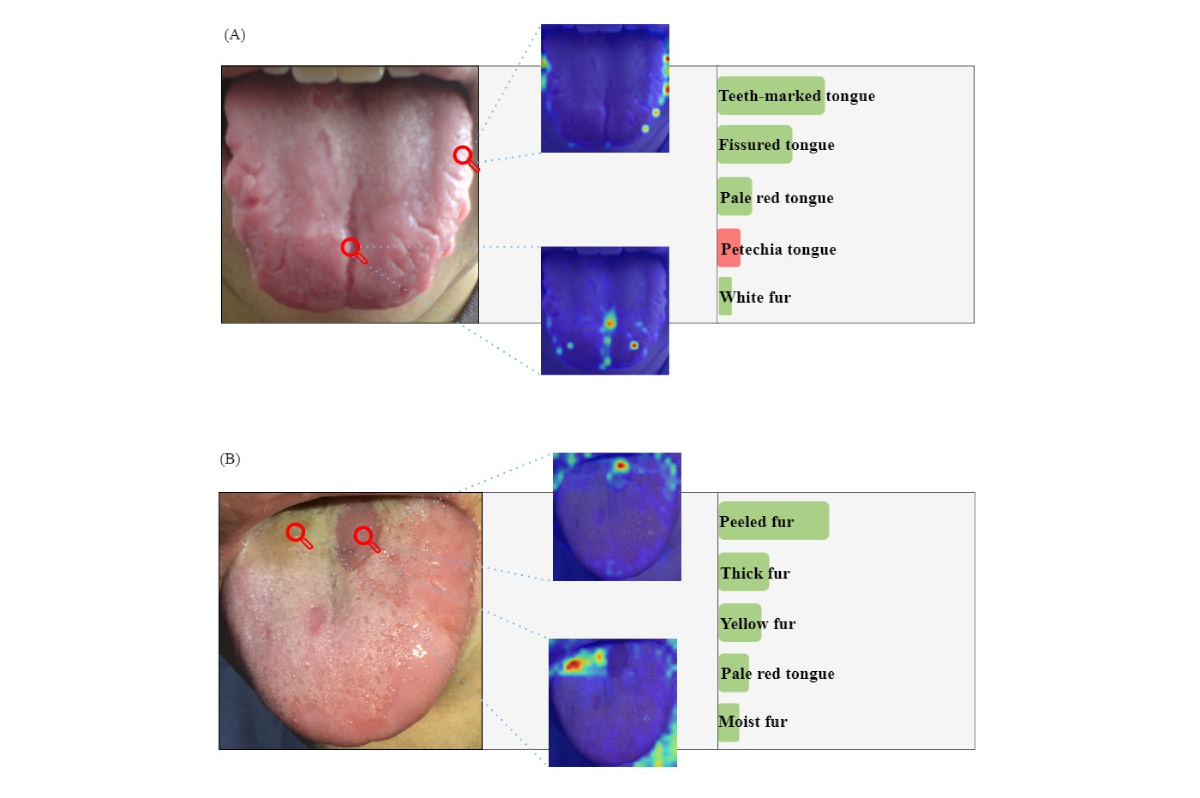
Visualization of the thermal response of the visual encoder’s multihead attention. Presents the top 5 categories and their confidence levels of tongue image features predicted by the model in text form. Green represents correctly predicted labels, red represents incorrectly predicted labels, and the length of the color bars intuitively indicates the probability of the prediction.

As shown in Figure 5, feature locations were selected in the tongue image; Figure 5A for fissured and teeth-marked tongues, and Figure 5B for peeled fur and yellow moss. The attention weights for the specified location are projected into the pseudo-color map of the original image. The text labels of the top 5 tongue features are obtained as output, with red indicating incorrect prediction and green indicating correct prediction, and the length of the color indicates the magnitude of its probability.

Through the feature attention visualization map, it can be seen that the visual encoder for tongue texture features focuses on localized regional positional responses, while the tongue color feature focuses on the overall area response of the image, so that the visual encoder can learn the effective feature information on the tongue, which provides good support for the subsequent feature fusion.

### Validation of Multimodal Capabilities Across Tasks

#### Tongue Image Description Generation Capability

In the task of describing the tongue images, we observe that LLaVA-1.6, LLaVA-OneVision, and Qwen2.5-VL-7B all generate output answers according to structured texts. Moreover, the text of LLaVA-1.6’s response to the tongue surface image was intermingled with the description of the tongue sublingual veins. This type of response may be a language hallucination of the LLM. The output text of the TongueVLM is relatively short, but in terms of content, it focuses mainly on image color, texture, and morphological information. Representative examples of model outputs are shown in Table S1 in Multimedia Appendix 1. The tongue feature interpretation of the TongueVLM model is closer to the results of visual model recognition, which fully demonstrates that the TongueVLM model, which has been fine-tuned with the tongue multimodal datasets, solves the conceptual alignment between the visual encoding of the images and the TCM terminology.

#### Physical Constitution Reasoning and Analysis Capability

In the task of physical constitution reasoning about the tongue image, we observe that the output of the LLaVA-1.5 model completely deviates from the TCM context and seems to lack an understanding of the concept of TCM physique, and the LLaVA-1.6 model outputs the reasoning thinking about TCM physique through the tongue image. The LLaVA-OneVision model misjudges the tongue feature as a red tongue, but can reason about the physical constitution in combination with the tongue feature. Of course, the output is also wrong. QWen2.5-VL-7B still follows some kind of templated output content, which is accurate in understanding the tongue color and color of the tongue fur features, but misclassifies the tongue shape as fat and large and dentate to the extent that the analysis of the physical constitution is not accurate enough.

In contrast, the TongueVLM model shows professional advantages in the TCM field in terms of effectively recognizing the tongue features and then briefly outputs the results through the tongue features, but its language expression level is slightly rough and needs to be further improved. Representative examples of model outputs are shown in Table S2 in Multimedia Appendix 1.

#### Tongue Image Dialogue Capability

In the multiround dialogue task of TCM with tongue images, we observe that all the models can accurately recognize the teeth-marked tongue feature in the tongue image in the first round of dialogue, and LLaVA-1.6 not only interprets the tongue feature dimension but also associates more scientific knowledge with the physical condition. However, in the second round of dialogue, LLaVA-1.5 and LLaVA-1.6 gave vague answers of “possibly related.” Moreover, LLaVA-OneVision and Qwen2.5-VL-7B were able to provide definite answers, but they were not related to the teeth-marked tongue feature in the previous round of dialogue. The TongueVLM model combines the content of the teeth-marked tongue feature and provides the exact answer; thus, the TongueVLM model still outperforms the other models. Representative examples of model outputs are shown in Table S3 in Multimedia Appendix 1.

The experimental results show that the TongueVLM model outperforms the general-purpose large model on all 3 tasks. First, in terms of tongue feature descriptions, the TongueVLM model is closest to the recognition results of the visual model and includes color and morphological features and local positional features, and the parallel computation time of multiple visual models is greater than that of the large model, which fully reflects the image feature retention ability and computational performance of the TongueVLM visual encoder. Second, in terms of physical constitution reasoning, it is obvious that LLaVA-1.5 does not have expertise in TCM. LLaVA-1.6, LLaVA-OneVision, and Qwen2.5-VL-7B are generated by interpreting the text according to the unified paradigm; it cannot distinguish between tongue photos and sublingual photos, and identifying the positive and negative features is difficult. The TongueVLM model, on the other hand, quantitatively analyzes and localizes the positive features of the tongue image and can reason about the physical constitution based on the tongue image features, with an accuracy of 78.6%. The traditional method requires multiple visual models to extract the tongue features and then input the feature data into decision trees, support vector machines, and other classifiers for supervised learning of somatic qualities, which is less efficient to execute. Third, in terms of multiround Q&As, the TongueVLM model’s interactive answers are brief, the contextual correlation is stronger, and the language discourse is not as strong as that of LLaVA-1.6, but its overall answers are more precise and concise, and language generation is not an advantage of the visual model.

## Discussion

### Principal Findings

This study proposes a TongueVLM network architecture design and a 3-stage training framework, achieving state-of-the-art performance on multimodal tongue image diagnosis tasks. Crucially, ablation experiments confirm that the staged training strategy is essential for stable optimization, while visual analysis demonstrates that the model spontaneously focuses on tongue regions relevant to clinical diagnosis.

The TongueVLM multimodal model proposed in this study is characterized by the following key features. First, the visual encoder characterizes the image features with the language encoding of low-dimensional vectors and approximates the feature extraction capability of the visual model by migration learning of TCM images from the CLIP-ViT pretrained model, which is a key step in the visual-to-large language model. Second, the modal fusion module splices visual and textual instruction data, realizes a seamless connection between natural language and image feature information, and unifies the multimodal data format input to the large model. Third, the TongueVLM model is fine-tuned and trained end-to-end on the TCM dataset. The model exhibits high accuracy in all 3 tasks of tongue feature description generation, physical constitution reasoning, and multiround dialogue.

### Limitations and Future Work

This study has several limitations. First, the current data modality only includes tongue image and text-based question-answering data, failing to encompass the complete information system of TCM’s “observation, auscultation, inquiry, and palpation.” Second, the dataset’s diversity requires enhancement, with shortcomings in ethnic distribution and standardization of image acquisition environments, which may compromise the model’s generalizability. Third, evaluation of generated texts (eg, tongue pattern descriptions) still relies on expert judgments with inherent subjectivity, lacking objective, fine-grained automated assessment criteria. Finally, the model’s generalization capability for clinically rare tongue patterns remains to be validated.

Future research may explore the following directions: first, in terms of model architecture, a gating mechanism is introduced to achieve adaptive attention regulation. Meanwhile, it is considered to incorporate modules such as depth-wise separable convolution to compensate for the shortcomings of self-attention mechanisms in capturing local detail features. Second, expand multimodal data sources by systematically integrating comprehensive information from facial diagnosis, pulse diagnosis, olfactory diagnosis, and physical constitution identification to construct a full-disease-course diagnostic model more aligned with TCM’s holistic perspective. Third, fuse multidimensional data acquisition technologies such as 400-1000 nm spectral data and thermal infrared imaging data, and 3D tongue morphology to establish a more refined quantitative tongue pattern characterization system. Fourth, explore automated evaluation frameworks based on LLMs to achieve fine-grained quantitative assessments of the professionalism, consistency, and logical coherence of generated content. Fifth, advance prospective trials in collaboration with clinical institutions to validate the model’s auxiliary diagnostic efficacy and clinical applicability in real-world diagnostic settings. Sixth, currently, TongueVLM is built upon a 7B-parameter model, and its deployment still imposes certain computational requirements. We will explore parameter-efficient fine-tuning techniques (eg, low-rank adaptation) and model quantization. Finally, the model will incorporate continuous learning mechanisms to iteratively improve its tongue image recognition accuracy and diagnostic reasoning ability, thereby advancing research on artificial intelligence–assisted TCM tongue diagnosis.

### Conclusions

Intelligent diagnosis and treatment technology in the field of TCM is often based on knowledge graphs, deep learning visual models, and machine learning approaches. Traditional supervised learning is limited by the quality and size of the training data, as well as the difficulty of fusion with natural language and its limited generalizability. The findings demonstrate that the TongueVLM model achieves ideal results in terms of understanding tongue images, constitutive reasoning, and dialogue abilities, and provides a foundational framework suitable for future integration into a Chinese medicine intelligent diagnosis system.

## Appendix

Multimedia Appendix 1 Tables (S1-S3) and figures (S1-S4), formulas, and example data.

## Abbreviations

API: application programming interface
CLIP‑ViT: Contrastive Language‑Image Pre‑Training With Vision Transformer
GELU: Gaussian Error Linear Unit
LLaMA: large language model meta artificial intelligence
LLM: large language model
Q&A: question and answer
TCM: traditional Chinese medicine
TCM-SFT: traditional Chinese medicine–supervised fine-tuning
VLM: visual language model

## Data Availability

The datasets generated and/or analyzed during this study are available from the corresponding author upon reasonable request.
