# Supplementary material for: Application of a Large Visual Language Model on Tongue Image Description Generation and Physical Constitution Reasoning in Traditional Chinese Medicine (TongueVLM): Model Development and Validation Study
Source: JMIR Med Inform. 2026 Mar 12;14:e87237. doi: 10.2196/87237 (PMC13022551; doi:10.2196/87237)
Supplement: Multimedia Appendix 1 [file medinform_v14i1e87237_app1.docx]

Table of Contents

A1.1 Tables S1-S3…………….........................………………......…………..….1

A1.2 Figures S1-S4………….........................……...………………….……..….4

A1.3 Tongue image descriptions dataset…………………...…………………..8

A1.4 Tongue image constitution reasoning dataset……………………...….….9

A1.5 Tongue image multi-round question and answer dataset……………...…10

A1.6 Visual encoder module Forward propagation process……………...….…11

A1.7 Modal fusion module Forward propagation process………….….……....12

A1.8 Language decoder module Forward propagation process……………......13

A1.9 Examples of tongue image comprehension skills in TCM…………..…...14

A1.10 Examples of tongue image somatic reasoning in TCM……….…...........17

A1.11 Example of tongue image dialogue skills in TCM……..……….............20

## A1.1 Supplementary Tables

**Table S1.** Representative examples of tongue image comprehension skills in TCM.


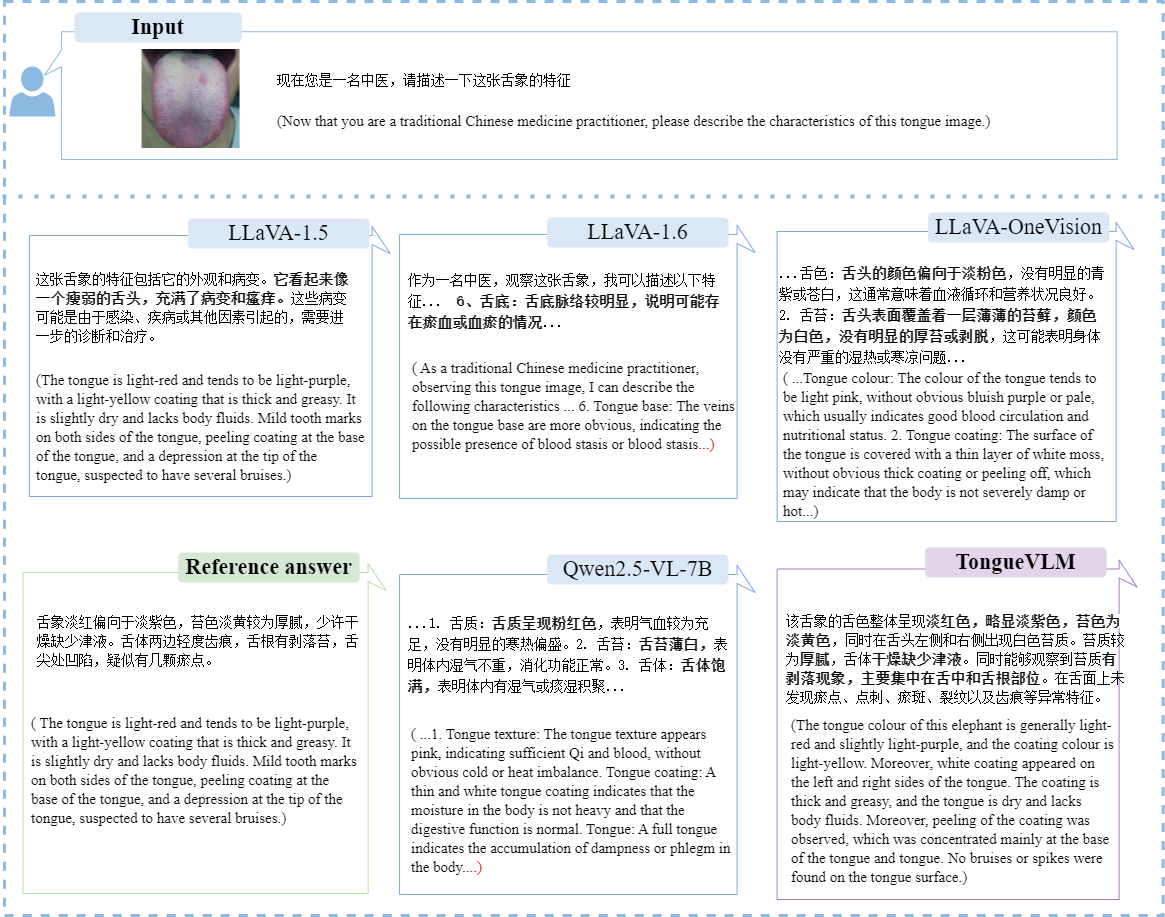


**Table S2.** Representative examples of tongue image somatic reasoning in TCM.


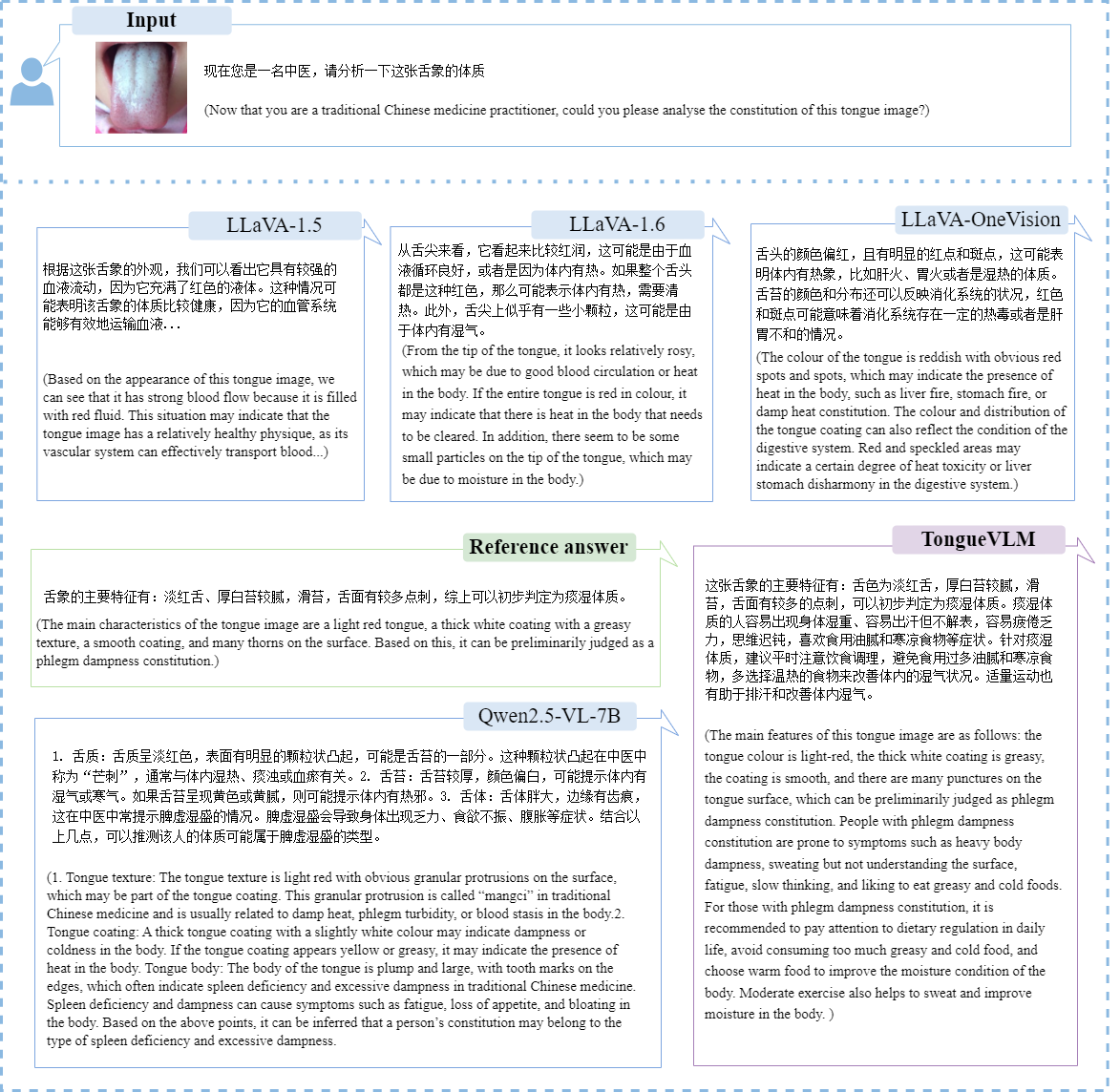


**Table S3.** Representative example of tongue image dialogue skills in TCM.


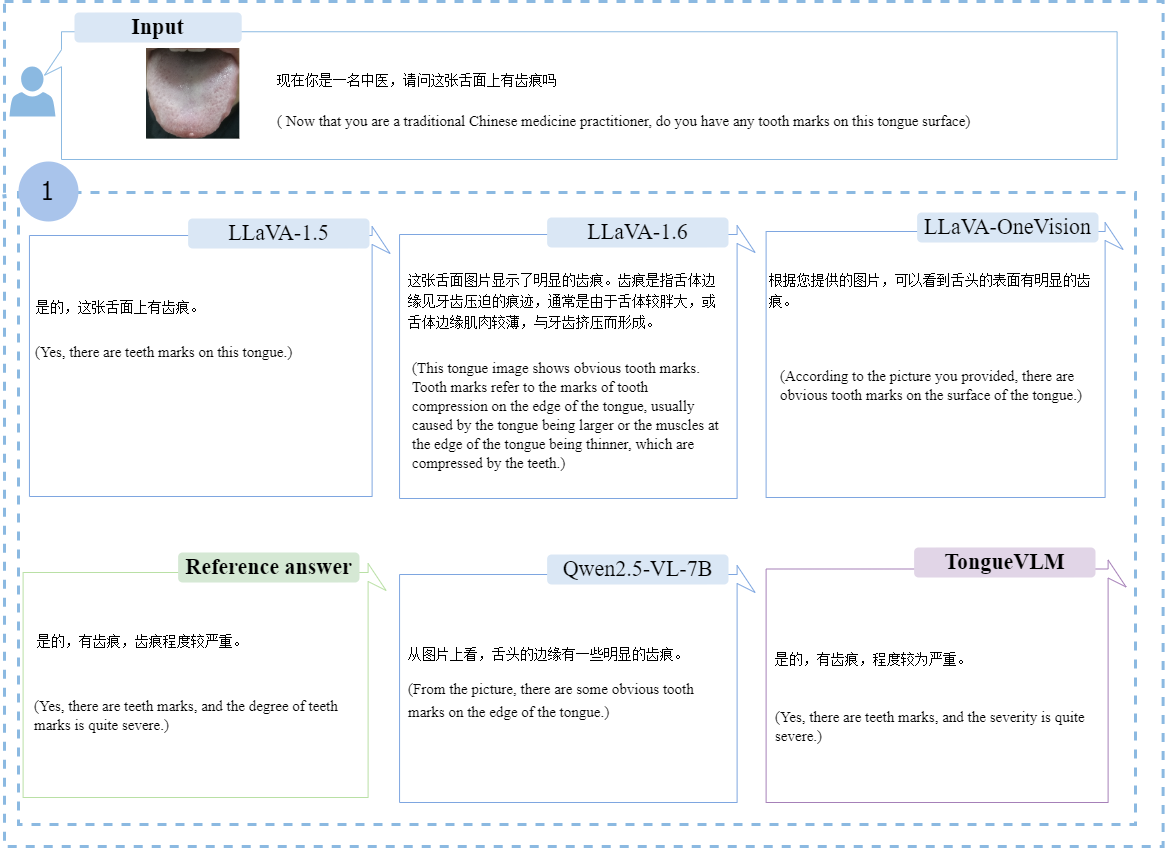


*
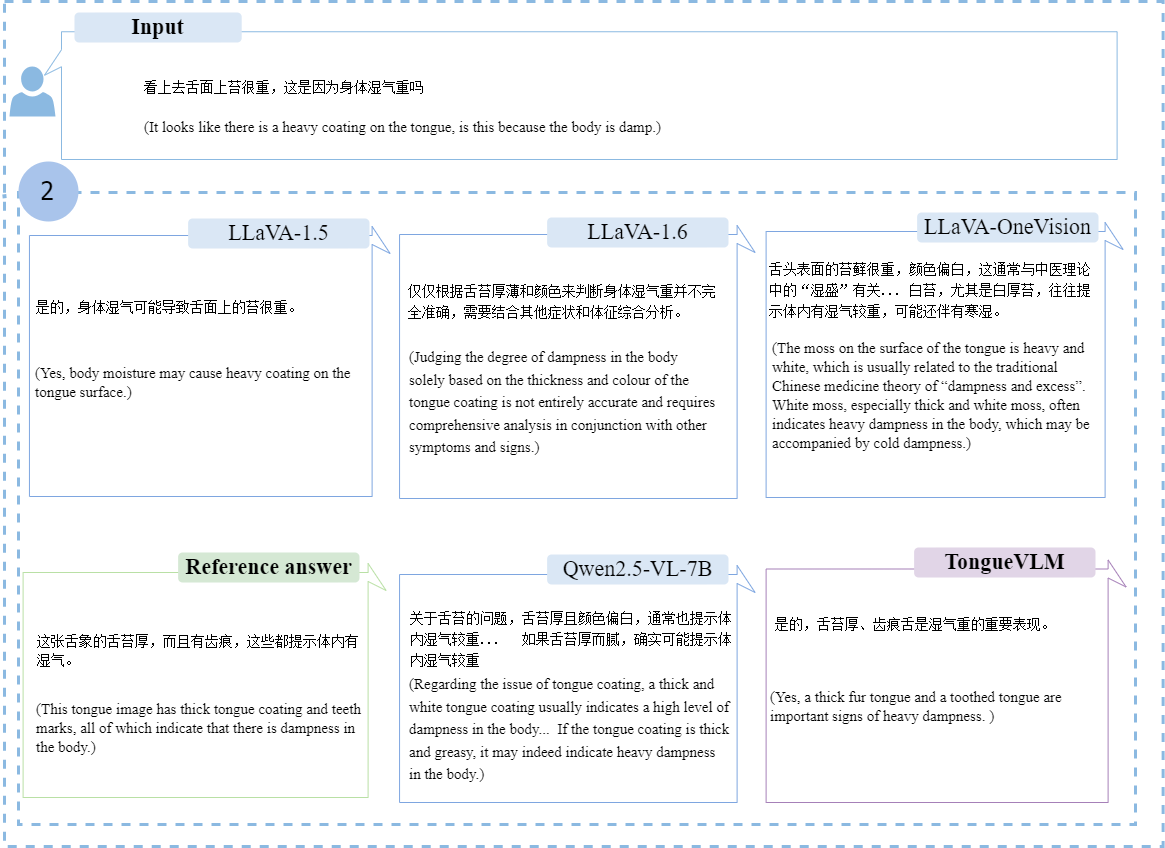
*

## A1.2 Supplementary Figures

**Figure S1.** Image patch and Transformer structure of the visual encoder.


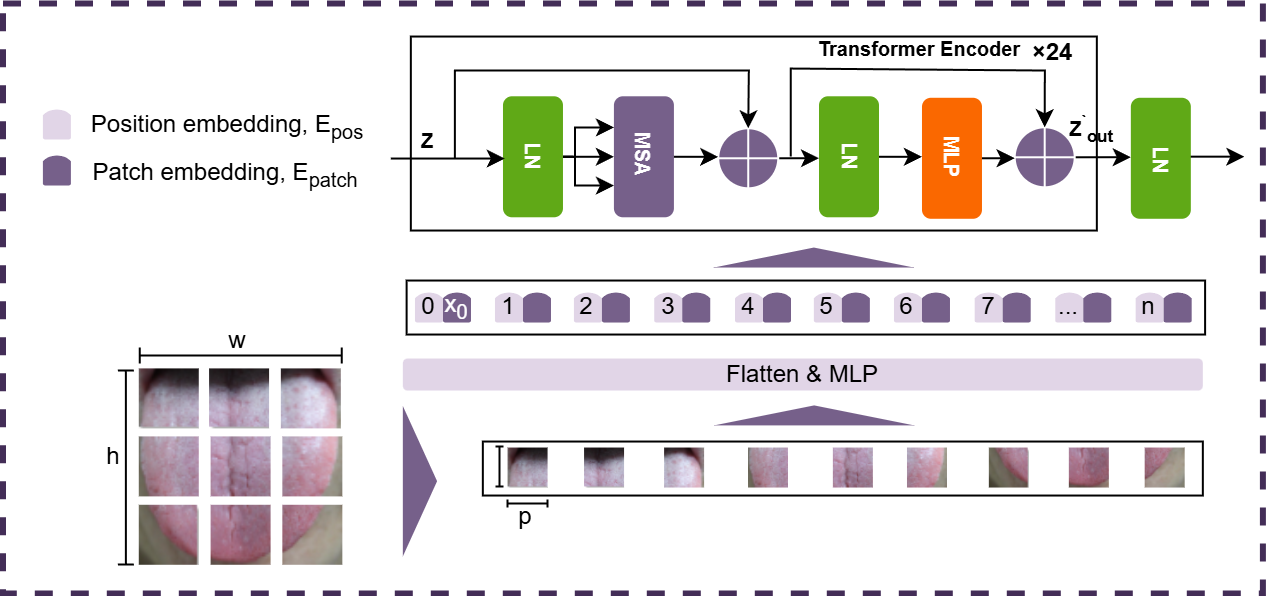


**Figure S2.** Design of the neural network unit for the TongueVLM.


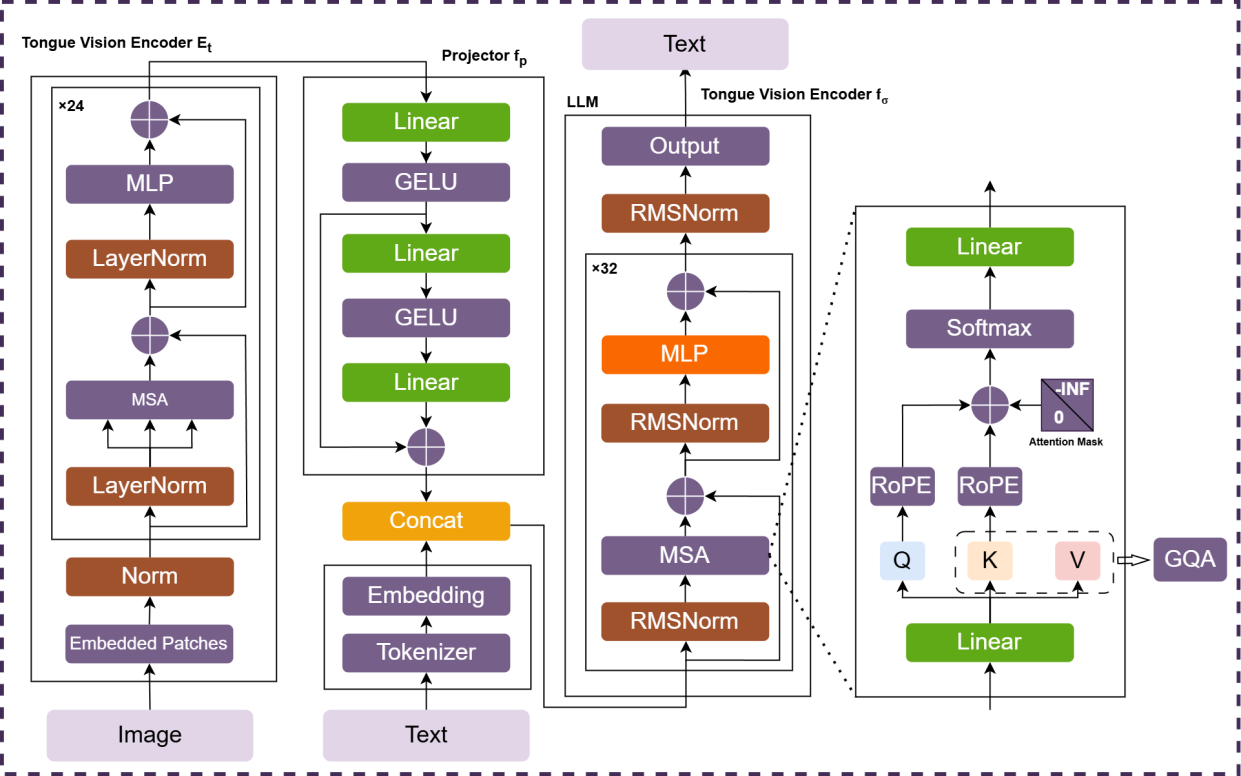


**Figure S3.** Distribution of feature values in the TCM tongue description dataset.


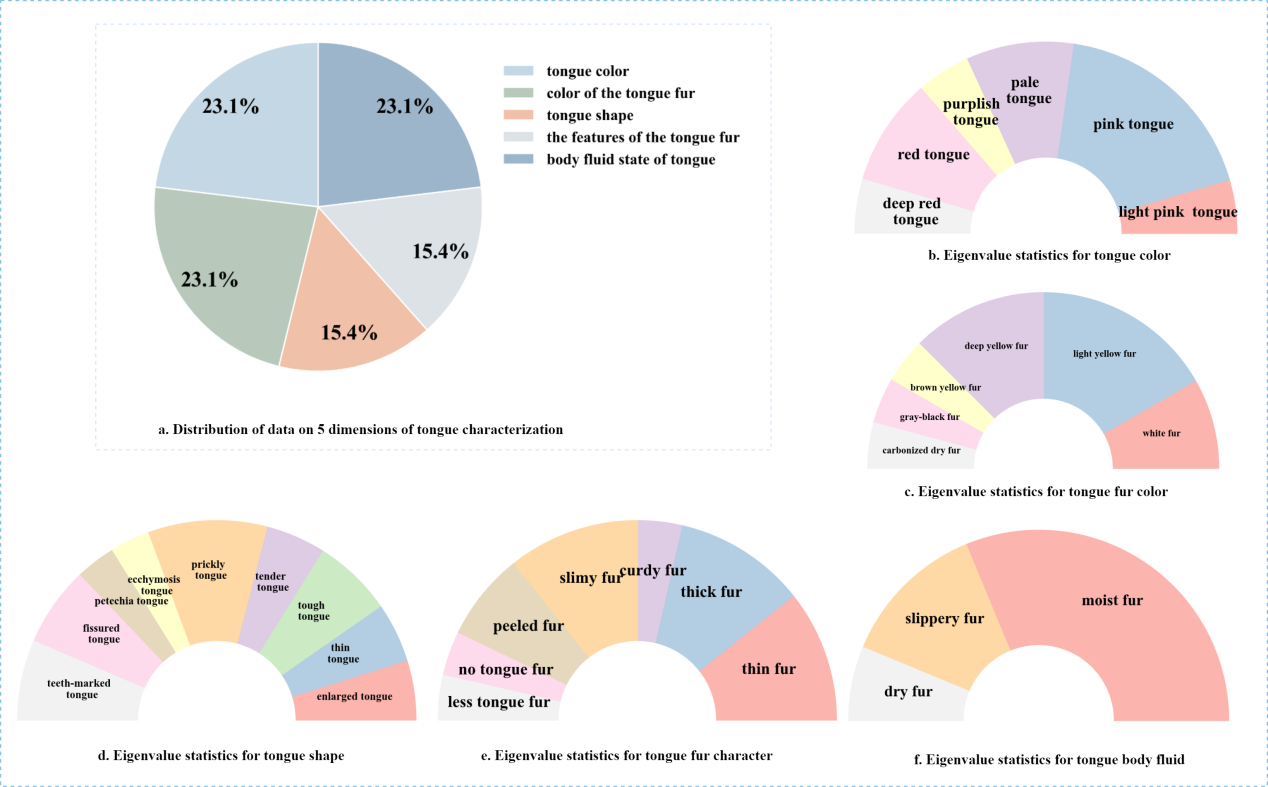


**Figure S4.** Collective mass distribution of TCM physical reasoning data.


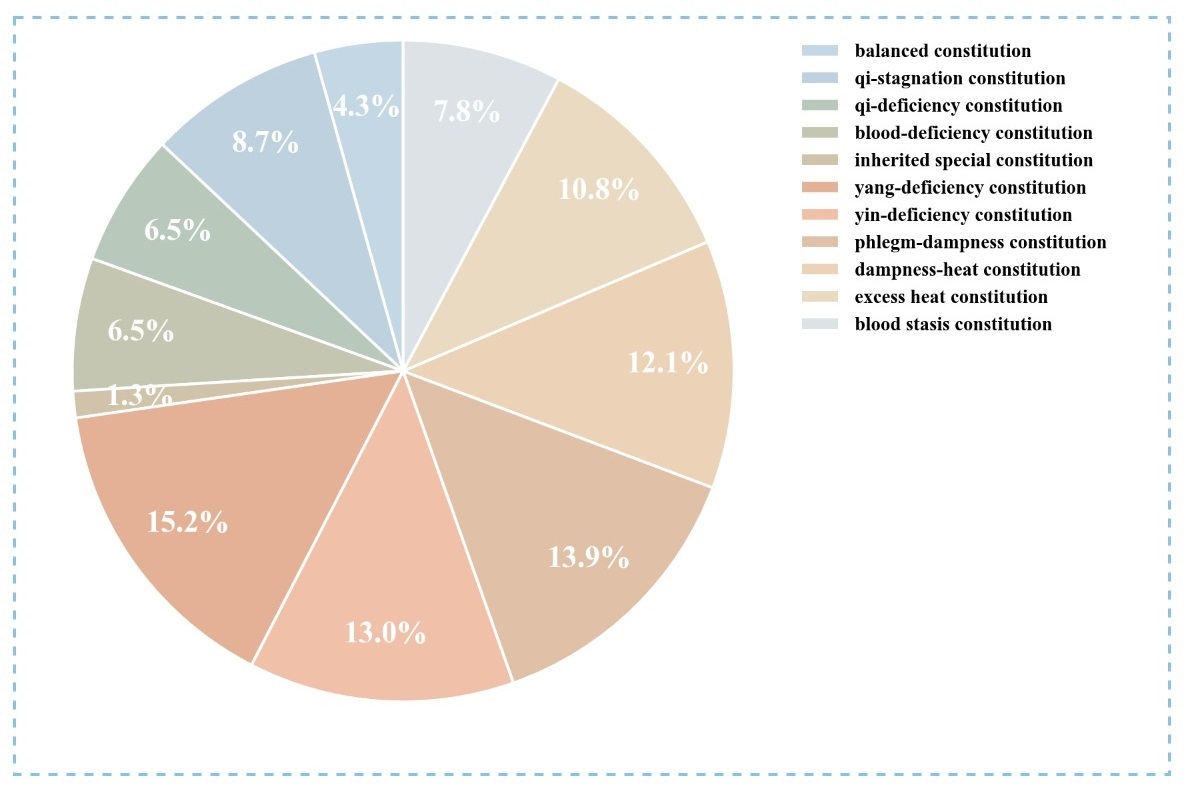


## A1.3 Tongue image descriptions dataset

For example, a tongue image with ID=1000006 has a TCM feature value C ∈ {pale red tongue, white fur, ecchymosis tongue, petechia tongue, thick fur, peeled fur}. Therefore, the following two multimodal datasets can be constructed for each tongue image map and feature value. (i) Tongue image and feature value text pair dataset$\text{<ID,}\text{C}\text{>}$, where ID represents the unique number of the image, C is a feature value of the tongue image, the total number of data records is 257,000, and the datasets are recorded as TCM-SFT-Characteristic-257k, of which the training set is 240,000, the validation set is 10,000, and the test set is 7,000. (ii) Tongue image and characterisation questions and their interpretations describing the dataset${\text{<ID,}\text{Q}_{\text{i}}\text{,D}}_{\text{i}}\text{>}$; ID represents the unique number of the image, $\text{Q}_{\text{i}}$ is an interrogation question about the tongue image features, $\text{D}_{\text{i}}$ is the professional interpretation for the interrogation question, and the total number of data records is 103,000; the dataset is recorded as TCM-SFT-Description-103k, of which the training set is 90,000, the validation set is 5,000, and the test set is 3,000. The distribution of the tongue image features in the dataset is shown in Figure S3.

## A1.4 Tongue image constitution reasoning dataset

To equip TongueVLM with the ability to analyse TCM constitution from tongue images, we produced a tongue image and constitution description text dataset $\text{<ID, Q,}\text{ }\text{H>}$. where ID represents the unique number of the image, $\text{Q}$ represents a question about the constitution inquiry, and H represents a professional interpretation of TCM constitution identification through tongue image. The total number of data records is 103,000, of which 90,000 are for the training set, 5,000 for the validation set, and 3,000 for the testing set; this dataset is denoted as TCM-SFT-Healthstatus-103k. The distribution of constitution qualities in the dataset is shown in Figure S4.

## A1.5 Tongue image multi-round question and answer dataset

Similarly, for TongueVLM to have the ability to interrogate tongue images in multiple rounds, we produced a dataset of multiple rounds of questions and answers $\text{<ID,\{\{}\text{Q}_{\text{1}}\text{,}\text{A}_{\text{1}}\text{\},...\{}\text{Q}_{\text{i}}\text{,}\text{A}_{\text{i}}\text{\}...,\{}\text{Q}_{\text{n}}\text{,}\text{A}_{\text{n}}\text{\}\}>}$(n is the number of rounds of conversation) about the tongue images, where ID stands for the unique number of the image and $\text{\{}\text{Q}_{\text{i}}\text{,}\text{A}_{\text{i}}\text{\}}$ is the i-th round of question $\text{Q}_{\text{i}}$ and answer $\text{A}_{\text{i}}$ of the conversation about the image. The total number of data records is 103,000, of which the training set is 90,000, the validation set is 5,000, and the test set is 3,000; this dataset is recorded as TCM-SFT-Communication-103k.

This method cumulatively yields 566,000 TCM multimodal datasets, of which the training set is 510,000, the validation set is 25,000, and the test set is 16,000, which can be used for the TongueVLM model for fine-tuning of each module. Twenty percent of the data were randomly selected from the dataset and calibrated by five TCM clinical practitioners.

## A1.6 Visual encoder module Forward propagation process

$$\begin{aligned} {\text{x}\text{=}\text{resize}\text{(x}}_{\text{v}}\text{)}\#\left( \text{3.1} \right) \end{aligned}$$

**where** $\text{x}_{\text{v}}\text{∈}\text{Z}^{\left( \text{H}\text{,}\text{W}\text{,}\text{C} \right)}\text{, }\text{x}\text{∈}\text{Z}^{\text{(h,}\text{w}\text{,}\text{C}\text{)}}$ $\text{,}\text{ h=}\text{w}\text{=336}$.

$$\begin{aligned} \text{x}_{\text{p}}\text{=}\text{patc}\text{h(}\text{x}\text{,}\text{p}\text{)}\#\left( \text{3.2} \right) \end{aligned}$$

**where** $\text{x}_{\text{p}}\text{∈}\text{R}^{\text{(}\text{N}^{\text{2}}\text{,}\text{p}^{\text{2}}\text{∙}\text{C}\text{)}}$ $\text{,}$ $\text{p}$=14.

$$\begin{aligned} \text{z}\text{=}\text{E}_{\text{patc}\text{h}}\text{+}\text{E}_{\text{pos}}\#\left( \text{3.3} \right) \end{aligned}$$

**where** $\text{z}\text{,}\text{E}_{\text{patc}\text{h}}\text{,}\text{E}_{\text{pos}}\text{∈}\text{R}^{\text{(}\text{N}^{\text{2}}\text{+1,}\text{dim}\text{)}}$ $\text{, }\text{dim}$=1024.

**where** $\text{E}_{\text{patch}}\text{=[}\text{x}_{\text{0}}\text{,projection(}\text{x}_{\text{p}}\text{,}\text{dim}\text{)]}$, $\text{x}_{\text{0}}\text{∈}\text{R}^{\text{dim}}$, $\text{E}_{\text{pos}}\text{=PE(}{{\text{[}\text{x}}_{\text{0}}\text{,}\text{x}}_{\text{p}}\text{],}\text{dim}$).

$$\begin{aligned} \text{z}_{\text{out}}{\text{=LN(MLP(LN(}\text{z}_{\text{l}}^{\text{'}}\text{))+}\text{z}}_{\text{l}}^{\text{'}}\text{) }\#\left( \text{3.4} \right) \end{aligned}$$

**where** $\text{z}_{\text{l}}^{\text{'}}\text{=MSA(LN(}\text{z}_{\text{l}\text{-1}}\text{))+}\text{z}_{\text{l}\text{-1}}$, $\text{ }\text{l}$=1... **L**, **L**=24, $\text{z}_{\text{out}}\text{∈}\text{R}^{\text{(}\text{N}^{\text{2}}\text{+1,}\text{dim}\text{)}}$.

$\text{z}_{\text{l}}^{\text{'}}$ is the MSA and MLP of the transformer encoder, which is repeated **L** times for encoding, and $\text{z}_{\text{out}}$ is the encoded output of the transformer.

## A1.7 Modal fusion module Forward propagation process

Splice visual encoding with textual word-embedded encoding to form a sequence of visual-textual instructions. First, the visual encoding feature $\text{Z}_{\text{v}}$ is converted into a visual feature vector $\text{H}_{\text{v}}$, and the textual instruction and textual description are converted into an instruction vector $\text{H}_{\text{q}}$ and textual description vector $\text{H}_{\text{c}}$ by word embedding encoding, respectively. Thereafter, the vectors are spliced in the order of instruction, visual, and textual description to form the visual-textual fusion instruction sequence data.

$$\begin{aligned} \text{H}_{\text{v}}\text{= }\text{Z}_{\text{v}}^{\text{'}}\text{+}\text{Z}_{\text{v}}^{\text{''}}\text{ }\#\left( \text{3.5} \right) \end{aligned}$$

**where** $\text{ }$ $\text{H}_{\text{v}}\text{∈}\text{R}^{\text{(}\text{N}^{\text{2}}\text{, }\text{dim}\text{)}}$, $\text{N}\text{=24}$, $\text{dim}\text{=4096}$

$\text{Z}_{\text{v}}^{\text{'}}$=$\text{GELU(Linear(}\text{E}_{\text{t}\text{, }\text{out}}\text{))}$,$\text{Z}_{\text{v}}^{\text{''}}\text{=Linear(GELU(Linear(}\text{Z}_{\text{v}}^{\text{'}}\text{)))}$

$$\begin{aligned} \text{H}_{\text{q}}\text{=}\text{text}_{\text{embedding}\left( \text{X}_{\text{q}} \right)}\text{ }\text{H}_{\text{q}}\text{∈}\text{R}^{\text{(seq\_q, dim)}}\#\left( \text{3.6} \right) \end{aligned}$$

$\begin{aligned} \text{H}_{\text{c}}\text{=}\text{text\_embedding}\text{(}\text{X}_{\text{c}}\text{), }\text{H}_{\text{c}}\text{∈}\text{R}^{\text{(}\text{seq}\text{\_}\text{c}\text{, }\text{dim}\text{)}}\#\left( \text{3.7} \right) \end{aligned}$

$$\begin{aligned} \text{f}_{\text{p}\text{, }\text{out}}\text{=}{\text{concat}\text{(}\text{H}}_{\text{q}}\text{,}\text{H}_{\text{v}}\text{,}\text{H}_{\text{c}}\text{) }\text{f}_{\text{p}\text{, }\text{out}}\text{∈}\text{R}^{\text{( }\text{seq}\text{\_}\text{q}\text{+}\text{N}^{\text{2}}\text{+}\text{seq}\text{\_}\text{c}\text{, }\text{dim}\text{)}}\#\left( \text{3.8} \right) \end{aligned}$$

where $\text{E}_{\text{t, out}}$ is the output of the visual encoder, $\text{X}_{\text{q}}$ is the instruction, $\text{X}_{\text{c}}$ is the text description, $\text{seq}\text{\_}\text{q}$ is the length of the instruction sequence, $\text{seq}\text{\_}$*c* is the length of the text description sequence, (3.5) is the transformation function of the visual feature encoding, (3.6) and (3.7) are the text word embedding encoding, and (3.8) is the multimodal instruction splicing, which is the output of the modal fusion layer.

## A1.8 Language decoder module Forward propagation process

$$\begin{aligned} \text{z}_{\text{l}}^{\text{'}}\text{=}\left\{ \begin{aligned} \text{ GQA}\left( \text{RMSNorm}\left( \text{f}_{\text{p}\text{, }\text{out}} \right)\text{,}\text{mask}_{\text{attention}} \right)\text{+}\text{f}_{\text{p}\text{, }\text{out}}\text{, }\text{l}\text{=1} \\ \text{GQA}\left( \text{RMSNorm}\left( \text{z}_{\text{l}\text{-1}} \right)\text{,}\text{mask}_{\text{attention}} \right)\text{+}\text{z}_{\text{l}\text{-1}}\text{,}\text{l}\text{>1} \end{aligned} \right.\#\left( \text{3.9} \right) \end{aligned}$$

$$\begin{aligned} \text{z}_{\text{out}}{\text{=MLP(RMSNorm(}\text{z}_{\text{l}}^{\text{'}}\text{))+z}}_{\text{l}}^{\text{'}}\text{ }\text{z}_{\text{out}}\text{∈}\text{R}^{\text{( seq\_q+}\text{N}^{\text{2}}\text{+seq\_c, }\text{dim}\text{)}}\#\left( \text{3.10} \right) \end{aligned}$$

$$\begin{aligned} \text{f}_{\text{σ}\text{, }\text{out}}\text{=RMSNorm(}\text{z}_{\text{out}}\text{) }\text{f}_{\text{σ}\text{, }\text{out}}\text{∈}\text{R}^{\text{( seq\_q+}\text{N}^{\text{2}}\text{+seq\_c, }\text{dim}\text{)}}\#\left( \text{3.11} \right) \end{aligned}$$

where $\text{l}$=1... L, **L**=32, the encoding is repeated **L** times for (3.9), GQA is the group attention mechanism, $\text{mask}_{\text{attention}}$ is the attention mask, RMSNorm is root-mean-square normalisation, and $\text{f}_{\text{σ, out}}$ is the output of the language decoder.

## A1.9 Examples of tongue image comprehension skills in TCM

The examples presented were carefully selected sampled from the test dataset can reflectthe model's typical performance.This example image has a particularly typical tongue appearance, as the tongue image has high specificity and rich diagnostic attributes (such as color, texture, coating quality), making it very suitable for testing and evaluating the model.

| **输入** | 现在您是一名中医，请描述一下这张舌象的特征？  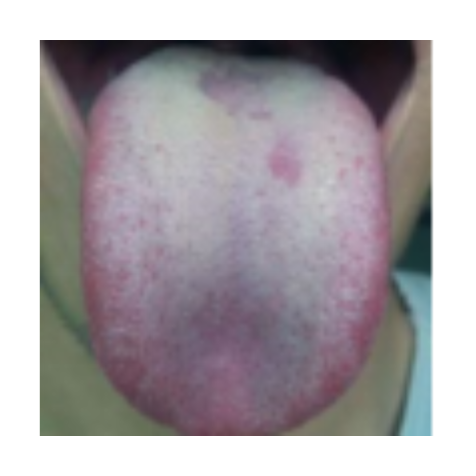 |
| --- | --- |
| **LLAVA-1.5** | 这张舌象的特征包括它的外观和病变。它看起来像一个瘦弱的舌头，充满了病变和瘙痒。这些病变可能是由于感染、疾病或其他因素引起的，需要进一步的诊断和治疗。 |
| **LLAVA-1.6** | 作为一名中医，观察这张舌象，我可以描述以下特征： 1、舌体：舌体较胖大，舌质较淡，舌面有齿痕，说明舌体可能较厚，舌质可能较淡，舌面有齿痕可能提示脾虚或水湿内停。 2、舌苔：舌苔较厚，颜色较白，说明可能有湿气或痰饮内停，也可能提示胃气不足。 3、舌色：舌色较淡，说明可能存在气血不足或阳虚的情况。 4、舌质：舌质较嫩，说明可能存在阴虚或气血不足的情况。 5、舌边：舌边有齿痕，说明可能存在脾虚或水湿内停的情况。 6、舌底：舌底脉络较明显，说明可能存在瘀血或血瘀的情况。 7、舌尖：舌尖较红，说明可能存在心火旺或心阴虚的情况。 8、舌中：舌中较淡，说明可能存在脾胃虚弱或气血不足的情况。 9、舌根：舌根较淡，说明可能存在肾阳虚或肾阴虚的情况。 10、舌面：舌面较光滑，说明可能存在津液不足或阴虚的情况。以上是根据这张舌象所观察到的特征，但需要注意的是，舌象只是中医诊断的一部分，还需要结合其他症状和体征，以及脉象、面色、问诊等综合判断，才能得出更准确的诊断。 |
| **LLAVA-OneVision** | 这张图片显示的是一张舌头的特写照片。从中医的角度来看，舌头可以反映人体的健康状况。以下是这张图片中舌头的几个特点： 1. 舌色：舌头的颜色偏向于淡粉色，没有明显的青紫或苍白，这通常意味着血液循环和营养状况良好。 2. 舌苔：舌头表面覆盖着一层薄薄的苔藓，颜色为白色，没有明显的厚苔或剥脱，这可能表明身体没有严重的湿热或寒凉问题。 3. 舌面纹理：舌头的纹理看起来比较细腻，没有明显的裂纹或瘀点，这可能意味着口腔黏膜健康，没有干燥或炎症的迹象。 4. 舌尖和舌根：舌头的尖端和根部没有明显的异常，这通常意味着心脏和肾脏的功能相对正常。 5. 舌边和舌根边缘：舌头的边缘和根部边缘没有明显的瘀点或紫斑，这可能表明没有肝郁气滞或瘀血阻滞的情况。 需要注意的是，中医的诊断是基于舌头的综合观察，包括舌头的颜色、纹理、苔藓的厚薄和分布、以及舌头的运动情况等。此外，中医诊断还需要结合患者的症状、体质、生活习惯等多方面信息。因此，这张图片只能提供舌头的一些基本信息，而无法做出确切的中医诊断。如果需要更准确的诊断，建议咨询专业的中医医生进行详细的望诊和辨证施治。 |
| **QWen2.5-VL-7B** | 从中医的角度来看，舌象是诊断疾病的重要依据之一。根据图片中的舌象特征，我们可以进行一些基本的分析： 1. 舌质：舌质呈现粉红色，表明气血较为充足，没有明显的寒热偏盛。 2. 舌苔：舌苔薄白，表明体内湿气不重，消化功能正常。 3. 舌体：舌体饱满，表明体内有湿气或痰湿积聚。 结合这些特征，可以推测该患者可能体质较虚，容易出现湿气或痰湿的情况。建议在中医师的指导下，通过中药调理、饮食调整和生活习惯改善来帮助身体恢复平衡。如果症状持续或加重，应及时就医。 |
| **TongueVLM** | 该舌象的舌色整体呈现淡红色，略显淡紫色，苔色为淡黄色，同时在舌头左侧和右侧出现白色苔质。苔质较为厚腻，舌体干燥缺少津液。同时能够观察到苔质有剥落现象，主要集中在舌中和舌根部位。在舌面上未发现瘀点、点刺、瘀斑、裂纹以及齿痕等异常特征。 |
| **Reference answer** | 舌象淡红偏向于淡紫色，苔色淡黄较为厚腻，少许干燥缺少津液。舌体两边轻度齿痕，舌根有剥落苔，舌尖处凹陷，疑似有几颗瘀点。 |

## A1.10 Examples of tongue image somatic reasoning in TCM

| **输入** | 现在您是一名中医，请分析一下这张舌象的体质？  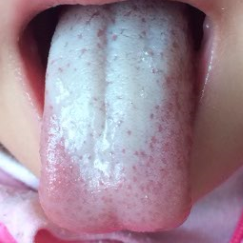 |
| --- | --- |
| **LLAVA-1.5** | 根据这张舌象的外观，我们可以看出它具有较强的血液流动，因为它充满了红色的液体。这种情况可能表明该舌象的体质比较健康，因为它的血管系统能够有效地运输血液。然而，需要注意的是，这只是一个简单的分析，并不能完全代表该舌象的整体健康状况。要确定舌象的健康状况，需要进行更全面的评估和检查。 |
| **LLAVA-1.6** | 作为一名中医，我需要观察舌象的多个方面来分析体质。然而，由于这张图片只显示了舌尖的部分，我只能根据这个部分提供一些初步的观察。从舌尖来看，它看起来比较红润，这可能是由于血液循环良好，或者是因为体内有热。如果整个舌头都是这种红色，那么可能表示体内有热，需要清热。此外，舌尖上似乎有一些小颗粒，这可能是由于体内有湿气。 |
| **LLAVA-OneVision** | 这张图片显示的是一张舌头的照片，舌头表面有明显的红点和斑点，这通常被称为“舌苔”。舌苔的颜色和分布可以提供一些关于人体健康状况的线索。在中医理论中，舌苔的颜色和分布与脏腑的阴阳寒热、湿热的状况有关。 从这张图片来看，舌头的颜色偏红，且有明显的红点和斑点，这可能表明体内有热象，比如肝火、胃火或者是湿热的体质。舌苔的颜色和分布还可以反映消化系统的状况，红色和斑点可能意味着消化系统存在一定的热毒或者是肝胃不和的情况。 |
| **QWen2.5-VL-7B** | 从中医的角度来看，舌象是诊断疾病的重要依据之一。根据图片中的舌象特征，我们可以进行一些基本的分析： 1. 舌质：舌质呈淡红色，表面有明显的颗粒状凸起，可能是舌苔的一部分。这种颗粒状凸起在中医中称为“芒刺”，通常与体内湿热、痰浊或血瘀有关。 2. 舌苔：舌苔较厚，颜色偏白，可能提示体内有湿气或寒气。如果舌苔呈现黄色或黄腻，则可能提示体内有热邪。 3. 舌体：舌体胖大，边缘有齿痕，这在中医中常提示脾虚湿盛的情况。脾虚湿盛会导致身体出现乏力、食欲不振、腹胀等症状。 结合以上几点，可以推测该人的体质可能属于脾虚湿盛的类型。 |
| **TongueVLM** | 这张舌象的主要特征有：舌色为淡红舌，厚白苔较腻，滑苔，舌面有较多的点刺，可以初步判定为痰湿体质。痰湿体质的人容易出现身体湿重、容易出汗但不解表，容易疲倦乏力，思维迟钝，喜欢食用油腻和寒凉食物等症状。针对痰湿体质，建议平时注意饮食调理，避免食用过多油腻和寒凉食物，多选择温热的食物来改善体内的湿气状况。适量运动也有助于排汗和改善体内湿气。 |
| **Reference answer** | 舌象的主要特征有：淡红舌、厚白苔较腻，滑苔，舌面有较多点刺，综上可以初步判定为痰湿体质。 |

## A1.11 Example of tongue image dialogue skills in TCM

|  | **第1轮** | **第2轮** |
| --- | --- | --- |
| **输入** | 现在你是一名中医，请问这张舌面上有齿痕吗？  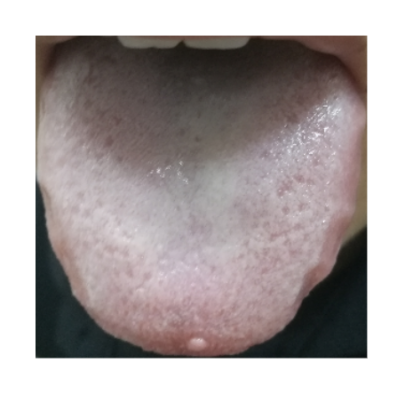 | 看上去舌面上苔很重，这是因为身体湿气重吗？ |
| **LLAVA-1.5** | 是的，这张舌面上有齿痕。 | 是的，身体湿气可能导致舌面上的苔很重。 |
| **LLAVA-1.6** | 这张舌面图片显示了明显的齿痕。齿痕是指舌体边缘见牙齿压迫的痕迹，通常是由于舌体较胖大，或舌体边缘肌肉较薄，与牙齿挤压而形成。 | 仅仅根据舌苔厚薄和颜色来判断身体湿气重并不完全准确，需要结合其他症状和体征综合分析。 |
| **LLAVA-OneVision** | 根据您提供的图片，可以看到舌头的表面有明显的齿痕。 | 舌头表面的苔藓很重，颜色偏白，这通常与中医理论中的“湿盛”有关。在中医看来，苔藓的厚薄和颜色可以反映身体的湿气状况。白苔，尤其是白厚苔，往往提示体内有湿气较重，可能还伴有寒湿。 |
| **QWen2.5-VL-7B** | 从图片上看，舌头的边缘有一些明显的齿痕。 | 关于舌苔的问题，舌苔厚且颜色偏白，通常也提示体内湿气较重。舌苔是舌面覆盖的一层白色或黄色的物质，它反映了体内湿气、热邪、寒邪等多种病理因素的存在。如果舌苔厚而腻，确实可能提示体内湿气较重。 |
| **TongueVLM** | 是的，有齿痕，程度较为严重。 | 是的，舌苔厚，齿痕舌是湿气重的重要表现。 |
| **Reference answer** | 是的，有齿痕，齿痕程度较严重。 | 这张舌象的舌苔厚，而且有齿痕，这些都提示体内有湿气。 |
